# PSMA-targeting hybrid molecule PSMA-914 for preoperative imaging and intraoperative fluorescence-guidance: A first-in-human experience

**DOI:** 10.7150/thno.129889

**Published:** 2026-07-05

**Authors:** Ann-Christin Eder, Mohamed A. Omrane, Jessica Matthias, Sven Stadlbauer, Mareike Roscher, Kerstin Michalski, Martin A. Schick, Christoph-Ferdinand Wielenberg, Peter Bronsert, Christian Gratzke, Martin Freitag, Klaus Kopka, Matthias Eder, Philipp T. Meyer, Cordula A. Jilg, Juri Ruf

**Affiliations:** 1 Department of Nuclear Medicine, Medical Center - University of Freiburg, Faculty of Medicine, University of Freiburg, Freiburg, Germany.; 2 Division of Radiopharmaceutical Development, German Cancer Consortium (DKTK), partner site Freiburg, Freiburg, Germany and German Cancer Research Center, Heidelberg, Germany.; 3 Department of Optical Nanoscopy, Max Planck Institute for Medical Research, Heidelberg, Germany.; 4 Division of Radiopharmaceutical Chemistry, German Cancer Research Center (DKFZ), Heidelberg, Germany.; 5 Helmholtz-Zentrum Dresden-Rossendorf (HZDR), Institute of Radiopharmaceutical Cancer Research, Dresden, Germany.; 6 TUD Dresden University of Technology, School of Science, Faculty of Chemistry and Food Chemistry, Dresden, Germany.; 7 Service Unit for Radiopharmaceuticals and Preclinical Studies, German Cancer Research Center (DKFZ), Heidelberg, Germany.; 8 Department of Anesthesiology and Critical Care, Medical Center–University of Freiburg, Faculty of Medicine, University of Freiburg, Freiburg, Germany.; 9 Department of Pathology, Medical Center - University of Freiburg, Faculty of Medicine, University of Freiburg, Freiburg, Germany.; 10 Department of Urology, Medical Center – University of Freiburg, Faculty of Medicine, University of Freiburg, Freiburg, Germany.; 11 German Cancer Consortium (DKTK), partner site Dresden, Dresden, Germany.; 12 German Cancer Consortium (DKTK), partner site Freiburg, Freiburg, Germany.

**Keywords:** prostate cancer, PSMA-targeting, hybrid molecule, fluorescence-guided surgery, PET/CT imaging

## Abstract

Surgery remains the cornerstone of prostate cancer treatment, demanding accurate tumor delineation and margin assessment. The hybrid molecule PSMA-914, which combines positron emission tomography (PET) and near-infrared fluorescence (NIRF) detection capabilities, was developed to improve preoperative imaging and intraoperative guidance during prostate cancer surgery.

**Methods:**

In this first-in-human series, prostate cancer patients underwent preoperative PET/CT imaging with ^68^Ga-labeled PSMA-914 (n = 10), followed by fluorescence-guided surgery after intraoperative PSMA-914 administration (n = 7, doses ranging from 200 -1500 µg/patient). The optimal dose for effective tumor visualization was retrospectively evaluated, and both preoperative and postoperative imaging results were correlated with histopathological findings.

**Results:**

Preoperative PET/CT with [^68^Ga]Ga-PSMA-914 showed high tracer uptake in primary tumors and metastases, facilitating accurate tumor localization. Intraoperatively, PSMA-914 at doses exceeding 1000 µg/patient provided a clear fluorescence signal, allowing for precise tumor and lymph node delineation. This approach significantly aided surgical resection by providing real-time guidance to differentiate malignant tissue from healthy tissue without altering the standard surgical workflow. Histopathological analysis confirmed the accuracy of [^68^Ga]Ga-PSMA-914 PET/CT staging. The procedure was well tolerated without adverse effects.

**Conclusion:**

The dual modality of PSMA-914 has the potential to significantly advance prostate cancer surgery by combining diagnostic imaging and intraoperative guidance. These first results warrant further large-scale clinical trials to validate efficacy and safety across diverse patient populations. The integration of PSMA-914 into clinical protocols promises to benefit prostate cancer surgery by facilitating real-time, high-contrast visualization to aid in complete tumor resection.

## Introduction

In case of localized and locally advanced prostate cancer, radical resection of the primary tumor including lymph node dissection with lymphadenectomy is an established curative strategy 1. Here, the precise detection and complete removal of all malign residues is crucial for a successful therapy outcome. Even in an oligo-metastatic stage, improvement of overall outcome (biochemical recurrence and overall survival) can be expected from a radical prostatectomy with removal of suspected lymph node metastases 2. Precise preoperative information on the primary tumor and metastases (e.g. lymph node metastases) can be obtained with cancer-targeting diagnostic radiopharmaceuticals for PET/CT imaging 3, 4. Via cognitive fusion, this information is transformed into surgical action. However, accurate intraoperative localization and delineation of PET-localized tumor margins or lymph node metastases is still a major challenge in oncological surgery, with, for example, small lymph node metastases often being macroscopically not clearly identifiable. Established intraoperative workarounds include an indiscriminate template-based removal of all lymphatic tissue with the drawback of increased morbidity following extended radical lymphadenectomy. Additionally, there is an enhanced risk of residual tumorous structures being overlooked, implying tumor recurrence and a high, potentially fatal burden for the patient. Although improvements can be achieved by combining PSMA-targeting radio-guided surgery (RGS) and radical prostatectomy 5-7, there is still an urgent need for advances in the field of intraoperative guidance.

For prostate cancer, first promising attempts to improve the intraoperative care of cancer patients included intraoperative radio- or fluorescence-based approaches 8, 9. Combining a radionuclide and near-infrared emitter in hybrid agents successfully united the strengths of both modalities 10, 11. However, first approaches were done in a sentinel-lymph node (SLN) concept only, which is based on the gradient-dependent lymphatic spread of the hybrid agent, and not the specific visualization of tumor tissue 12, 13. Due to the regional application of the SLN-technique, the high agent concentration close to the injection site leads to an increased background signal in the surrounding area so that lesions outside the distribution route might not be identified 11.

In contrast, cancer-specific hybrid molecules promise to overcome these limitations by supporting both non-invasive imaging-based diagnostics and surgery planning (e.g. PET/CT) based on the radioactive label, and subsequent visual intraoperative guidance based on the fluorescent label. By combining preoperative patient stratification and the therapeutic approach of guided-surgery in one molecule, this hybrid technology simplifies clinical translation and application by following a theranostic concept (diagnostics+therapy), which is of increasing importance in PCa management 14.

Based on the FDA-approved radiopharmaceutical [^177^Lu]Lu-PSMA-617 (^177^Lu-vipivotide tetraxetan, Pluvicto) for the treatment of prostate cancer, we recently developed specific hybrid molecules targeting the prostate-specific membrane antigen (PSMA) 15-17. The peptidomimetic low molecular weight format of this platform has proven to be advantageous in clinical application leading to FDA approval. Due to the fast pharmacokinetic (PK) profile, high tumor penetration, and high tumor retention, background accumulation in healthy tissue is low and severe side effects are minimized, rendering this therapy safe with low toxicity. The novel PSMA-specific hybrid molecules of this platform translate these advantageous molecular features in the framework of a theranostic application to surgical interventions with cancer-specific intraoperative guidance.

Advancing from a previous preclinical evaluation 16, we here report first experiences with preoperative PSMA-PET/CT imaging and subsequent fluorescence-guided surgery with the peptidomimetic PSMA-targeting hybrid molecule Glu-urea-Lys-(HE)_3_-HBED-CC-IRDye800CW (PSMA-914). This clinical translation evaluates the treatment of prostate cancer with a cancer-specific, low molecular weight, hybrid approach for preoperative imaging and intraoperative guidance.

## Materials and Methods

### Patients

Preoperative PET/CT imaging and fluorescence-guided resection with [^68^Ga]Ga-PSMA-914 was offered to selected patients with high-risk prostate cancer according to D’amico-classification 18. This was done in accordance with the updated Declaration of Helsinki, paragraph-37 “Unproven Interventions in Clinical Practice,” and in accordance with German regulations for medical indication of an unapproved drug in patients, in particular with regard to German pharmaceutical act §13(2b). Patients were informed about the experimental nature of this theranostic approach and gave written informed consent. The local ethical committee approved the retrospective evaluation as an observational study (#22-1057_1-retro).

### PSMA-914

Glu-urea-Lys-(HE)_3_-HBED-CC-IRDye800CW (PSMA-914) is a PSMA-targeting peptidomimetic hybrid molecule featuring a PSMA-binding moiety, a chelator for radiolabeling and IRDye800CW as a fluorescent-tag for near-infrared fluorescence imaging (excitation_max_ 774 nm/emission_max_ 789 nm) 16, 19. For PET/CT imaging PSMA-914 was labeled with ^68^Ga. For fluorescence imaging only, lyophilized PSMA-914 was reconstituted with sterile 0.9 % NaCl for injection. Details on toxicity and safety data as well as the radiopharmaceutical production of [^68^Ga]Ga-PSMA-914 are provided in the supplementary material (Supplemental Methods, Figure S1) 20, 21.

### PET/CT imaging protocol and analysis

Whole-body PET scans were acquired at 1 h and 2 h post-injection (p.i.) in 8 patients, and at 1 h p.i. in 2 patients, following administration of 118-217 MBq [^68^Ga]Ga-PSMA-914 (30 µg). Imaging was performed from mid-thigh to the base of the skull using a scan duration of 2 minutes per bed position. A low-dose CT (120 kVp, 25 mAs) was performed for attenuation correction and anatomic correlation. Scans were acquired on a VEREOS Digital PET/CT (Philips Healthcare, USA). Images were reconstructed with a vendor-specific time-of-flight iterative reconstruction algorithm (BLOB-OS-TF) with 3 iterations and 9 subsets (relaxation parameter 0.35) and a voxel size of 2 x 2 x 2 mm³ (VEREOS Digital PET/CT). The spatial resolution of the reconstructed PET image is about 5 mm full width at half maximum. PET scans were analyzed by two readers (KM, CFW) using the software PMOD (PMOD Technologies LLC, Zurich, Switzerland). The mean standardized uptake values (SUV_mean_) of the right parotid gland, right submandibular gland, right lung, mediastinal blood pool, right liver lobe, spleen, pancreas tail, small intestine, colon, voided bladder, right kidney, skeletal muscle (gluteus maximus left) and adipose tissue (adipose tissue of the left gluteal region) were analyzed 1 and 2 h p.i. With regard to the intestine, SUV was measured at the localization with the visually highest radiotracer uptake. For calculation of SUV_mean_, a 2 - 3 cm-diameter spherical volume of interest was drawn and all voxels at a 70 % isocontour were included. Furthermore, maximum SUV (SUV_max_) was assessed in lesions that were visually considered as suggestive of prostate cancer or metastases. Lymph node and bone metastases were analyzed separately, and the lymph node or the bone metastasis with the highest SUV_max_ was used for further comparisons.

### Surgical techniques and fluorescence-guided detection

Radical resection of primary tumors, including lymph node dissection or lymphadenectomy surgery, was performed 1-46 days after PET/CT imaging (for anesthesia and safety protocols, see Supplemental Information). The surgical strategy for tumor resection was based on the combined information obtained from multiparametric MRI, conventional CT imaging, and preoperative PSMA PET/CT, as well as intraoperative fluorescence guidance. As the majority of patients presented with locally advanced prostate cancer, a nerve-sparing approach was not pursued; instead, a wide excision including resection of the neurovascular bundles was performed, accompanied by an extended pelvic lymph node dissection. Intraoperative fluorescence imaging served as an additional real-time guidance tool without altering the predefined surgical approach. Both, open retropubic and minimal-invasive robotic assisted (DaVinci system) radical prostatectomy with extended lymph node dissection were performed state of the art as a part of the clinical routine. For fluorescence imaging, the hybrid molecule PSMA-914 was administered i.v. 1 h prior to surgery (between 200 µg and 1500 µg/patient, please refer to Table 1). For fluorescence detection during open radical prostatectomy, a conventional endoscopic camera with an appropriate light source (VisionSense VSiii3 3DHD IR Fluorescence Vision System) was used. The system comprised a 300-watt xenon-based illumination source (LS300) and a laser light source (LLS805-Laser; excitation wavelength: 785 nm). During robotic prostatectomy (DaVinci X® and Xi®) the integrated fluorescence imaging capability provided real-time near-infrared guidance (Excitation laser light source 803 nm, signal from fluorescence ICG 830 nm). The resected tissue samples were additionally examined for fluorescence signal *ex situ* using the conventional endoscope camera. All collected tissue samples subsequently underwent the standard of-care histopathological evaluation.

### Pathology

Prostate specimens and resected lymph nodes (LN) were grossly sectioned according to national guideline protocols after 24h formalin fixation in 10 % buffered formalin. LNs ≤ 4 mm in diameter were entirely embedded. LNs > 4 mm were macroscopically examined and suspicious areas were embedded. In the absence of macroscopically suspicious findings, one representative cross-section of the whole lymph node was embedded. Paraffin-embedding was conducted according to routine protocols. Next, from the paraffin-embedded tissue specimen serial tissue sections of 3 µm thickness (for Hematoxilin and Eosin (HE) staining and prostate-specific membrane antigen (PSMA) immunohistochemistry) were sliced using the Leica RM2255 Microtome. HE staining was automatically conducted using the Dako Cover Stainer.

Histopathological diagnosis was conducted by a board-certified pathologist. TNM classification and Gleason grading were performed in accordance with international standards.

For PSMA immunohistochemistry, all slides were incubated at 58 °C for two days in a drying chamber, then deparaffinized using xylene and rehydrated with graded ethanol series. Next, tissue specimens were stained using PSMA Ready-to-use antibody (IR089, Clone 3E6). EnVision® Flex Peroxidase-Blocking Reagent (DAKO, SM801) was first applied to block endogenous peroxidase activity, followed by incubation with EnVision® Flex+ Mouse LINKER (DAKO, K8021) to enhance primary antibody binding. EnVision® Flex/HRP (DAKO, SM802) conjugated with horseradish peroxidase was used as the secondary detection system. Counterstaining was performed via hematoxylin staining prior to mounting the slides with coverslips. The pathologist was blinded for PET-findings.

### Postoperative fluorescence imaging

Subsequently, formalin-fixed and paraffin-embedded tissue slices (10 µm) were macroscopically analyzed for PSMA-914 fluorescence using the Odyssey CLx system (LI-COR Biosciences, excitation wavelength 800 nm).

For visualizing PSMA-914 fluorescence on the microscopic level, confocal data were collected on a custom-built STED (Stimulated Emission Depletion) system similar to the one published by Gorlitz *et al*. 22. Details on the system, image acquisition and quantitative data analysis are described in the supplemental methods 23.

## Results

### Patients

A total of 10 patients (mean age at primary diagnosis: 65.1 years) with histologically confirmed prostate adenocarcinoma were included and underwent preoperative PET/CT with [^68^Ga]Ga-PSMA-914 (Tables 1 and 2). Among them, 8 patients had ISUP grade 5, 1 patient had ISUP grade 4 and 1 patient had ISUP grade 2 at biopsy. The mean initial PSA was 32.6 ng/ml (median: 11.3 ng/ml).

Prior to [^68^Ga]Ga-PSMA-914 PET/CT, all patients received at least one conventional imaging modality as part of their diagnostic work-up. This included six CT scans of the abdomen, six multiparametric MRI (mpMRI) of the prostate, and seven bone scintigraphy scans. These examinations were performed at different time points as part of pre-therapeutic staging. CT imaging revealed a range from no detectable prostate pathology (cN0, cM0) to suspected lymph node involvement and locally advanced prostate cancer (e.g., cT3b, cN1, cM1a/b) (Table 1). Multiparametric MRI (mpMRI) showed PI-RADS 5 lesions in several patients, with extracapsular extension and seminal vesicle invasion in some cases. Bone scans were performed in seven patients, mostly revealing degenerative changes; however, one scan indicated suspected bone metastases (cM1b). Overall, conventional imaging suggested locally advanced prostate cancer, with suspicion of regional lymph node metastases and, in some cases, distant metastases. These findings revealed heterogeneous tumor stages and served as the basis for the decision to perform further evaluation using PET/CT with [^68^Ga]Ga-PSMA-914. Of the 10 patients undergoing [^68^Ga]Ga-PSMA-914 PET/CT, 9 proceeded to surgery, while 1 patient deemed inoperable due to extensive metastatic disease.

Among the 9 patients who underwent surgery, 7 received additional cold PSMA-914 for fluorescence imaging. Patient no. 1 underwent surgery using the residual tracer from the preoperative PET/CT administered the day before surgery (30 µg). For Patient no. 4 cold PSMA-914 administration was omitted due to a technical failure of the intraoperative fluorescence imaging system on the day of surgery.

### Toxicity data and safety

Prior to the first-in-human use, a toxicity and dosimetry study was conducted in mice in order to assess the non-clinical safety profile, the tolerability and pharmacokinetics of PSMA-914. The subchronic toxicity study of PSMA-914 in male mice revealed no adverse effects at doses up to 2 mg/kg body weight, establishing a high safety margin for clinical use. The radiation dosimetry study indicated that [^68^Ga]Ga-PSMA-914 is predominantly cleared via the renal system, with an effective dose of 2.25 mSv for a standard diagnostic activity, supporting its safe application in PET imaging (details provided in the Supplemental Information). After injection of the cold ligand for surgical use, no adverse reactions were observed.

### Preoperative [^68^Ga]Ga-PSMA-914 PET/CT imaging identifies primary tumors and metastasis

**Figure 1:**
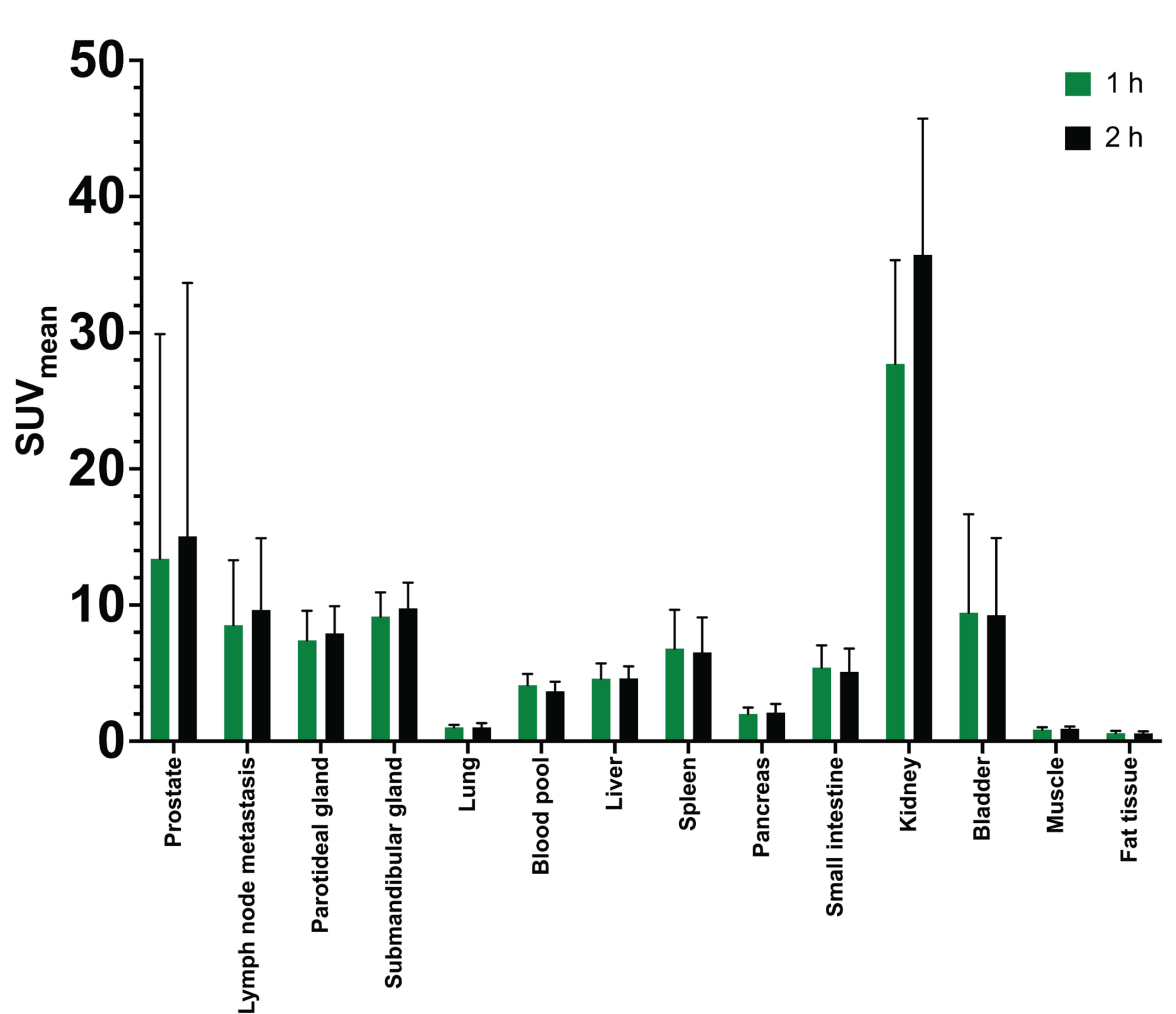
Bar plots of SUV_mean_ values showing tumorous and physiological tracer distribution of [^68^Ga]Ga-PSMA-914 PET. Dark green bars represent the values 1 h after injection, while black bars show the values 2 h after injection. Significant changes in organ distribution at 2 h p.i. as compared to 1 h p.i. were found only for the SUVmean of the submandibular glands (SUVmean at 1 h p.i. 9.2 ± 1.8 g/mL; SUVmean at 2 h p.i. 9.8 ± 1.9 g/mL; p < 0.01), the mediastinal blood pool (SUVmean at 1 h p.i. 4.1 ± 0.8 g/mL; SUVmean at 2 h p.i. 3.7 ± 0.7 g/mL; p = 0.04) and the kidneys (SUVmean at 1 h p.i. 27.7 ± 7.6 g/mL; SUVmean at 2 h p.i. 35.7 ± 10.0 g/mL; p < 0.01).

**Figure 2:**
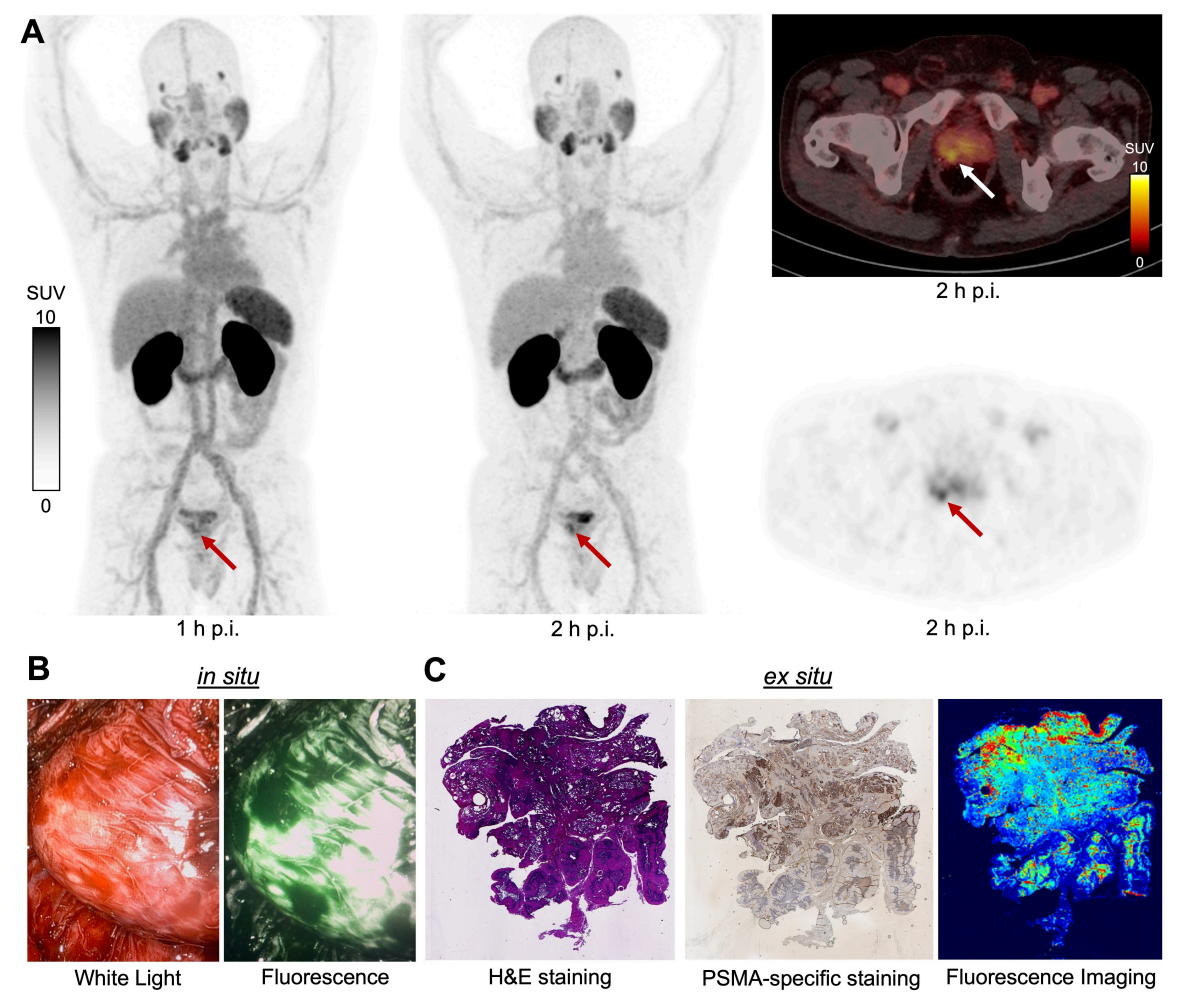
**Patient example of [^68^Ga]Ga-PSMA-914 PET/CT and subsequent robot-assisted radical prostatectomy.** (A) PET/CT maximum intensity projections at 1 and 2 h after injection of [^68^Ga]Ga-PSMA-914 (left and middle panels) with transaxial PET/CT fusion (right panel) and attenuation-corrected PET (lower right) at 2 h post-injection (red and white arrows indicating the primary tumor). (B) Intraoperative real-time fluorescence imaging of the cancerous prostate (1000 µg PSMA-914) under white light (left) and fluorescence (right). (C) Ex vivo analysis of resected tumor tissue (prostate): from left to right H&E staining, PSMA-specific immunohistochemical staining, and Odyssey CLx fluorescence imaging.

**Figure 3:**
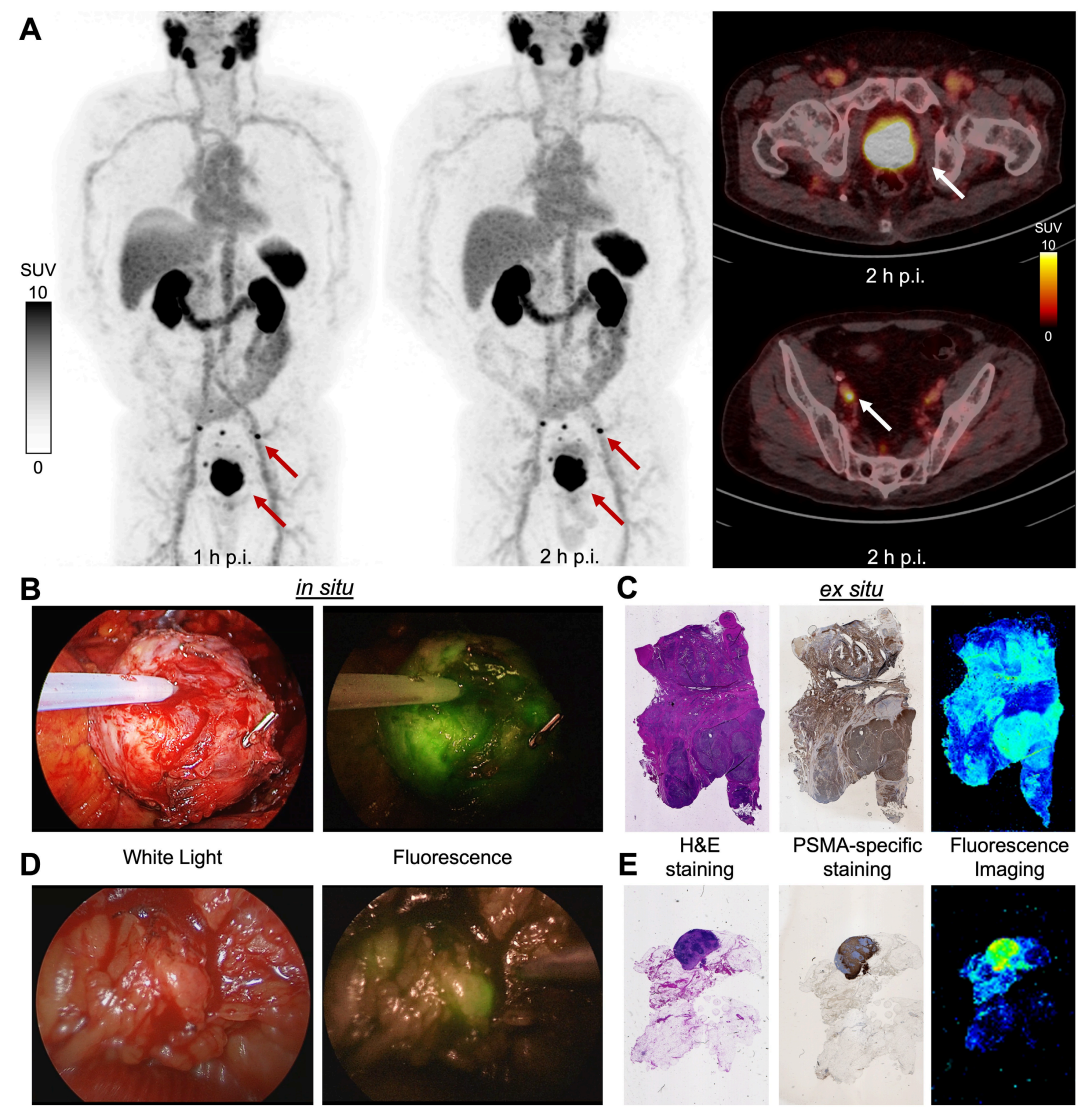
**Patient example of [^68^Ga]Ga-PSMA-914 PET/CT and retropubic open prostatectomy with lymph node dissection.** (A) PET/CT maximum intensity projections at 1 and 2 h after injection of [^68^Ga]Ga-PSMA-914 (left and middle panels) with transaxial PET/CT fusion (upper and lower right panel) at 2 h post-injection (red and white arrows indicating the primary tumor and lymphnode metastases). (B) Intraoperative real-time fluorescence imaging of the cancerous prostate (1000 µg PSMA-914) under white light (left) and fluorescence (right). (C) Ex situ analysis of resected tumor tissue (prostate): from left to right H&E staining, PSMA-specific immunohistochemical staining, and Odyssey CLx fluorescence imaging. (D) Intraoperative real-time fluorescence imaging of the metastatic lymph nodes (1000 µg PSMA-914) under white light (left) and fluorescence (right). (E) Ex situ analysis of resected tumor tissue (lymph node): from left to right H&E staining, PSMA-specific immunohistochemical staining, and Odyssey CLx fluorescence imaging.

Preoperative PET/CT imaging with [^68^Ga]Ga-PSMA-914 revealed high tracer uptake in the primary tumors at 1 h p.i. (mean SUV_max_ 16.3 ± 18.2 g/mL; range 5.9 – 65.2 g/mL) (Figure 1). Among the 8 patients with scans at 1 h and 2 h p.i. uptake in the primary tumor increased slightly at 2 h p.i. (mean SUV_max_ 1 h p.i.: 16.2 ± 20.2 vs. 2 h p.i.: 18.2 ± 22.3 g/mL), but changes were not significant (p = 0.15). Lymph node metastases (0 to 45 per patient) were found in six patients (mean SUV_max_ at 1 h p.i. 13.0 ± 8.2 g/mL, range 4.2 – 26.0 g/mL). Three patients presented with bone metastases (meanSUV_max_ at 1 h p.i. 10.0 g/mL, range 4.7 – 14.3 g/mL). High physiological tracer uptake was found in salivary glands, liver, spleen, small intestine and kidneys (Figures 1-3). Additionally, the images at 1 h and 2 h p.i. showed high blood pool activity (Figure 1-3). As expected, due to its hydrophilic profile, the tracer was excreted via the urinary system. Significant changes in organ distribution at 2 h p.i. as compared to 1 h p.i. were found only for the SUV_mean_ of the submandibular glands (SUV_mean_ at 1 h p.i. 9.2 ± 1.8 g/mL; SUV_mean_ at 2 h p.i. 9.8 ± 1.9 g/mL; p < 0.01), the mediastinal blood pool (SUV_mean_ at 1 h p.i. 4.1 ± 0.8 g/mL; SUV_mean_ at 2 h p.i. 3.7 ± 0.7 g/mL; p = 0.04) and the kidneys (SUV_mean_ at 1 h p.i. 27.7 ± 7.6 g/mL; SUV_mean_ at 2 h p.i. 35.7 ± 10.0 g/mL; p < 0.01). Figure 1 shows a comparison of the SUV_mean_ values after 1 h and 2 h after injection.

Preoperative assessment of the clinical TNM stage using PET/CT with [^68^Ga]Ga-PSMA-914 was highly concordant with the final pathological TNM classification. Correlating PET/CT staging with histopathology, PET/CT with [^68^Ga]Ga-PSMA-914 classified the nodal staging of all patients correctly (Table 1). In 9 patients undergoing surgery, 5 patients were prospectively diagnosed cN1 on PET/CT and subsequently confirmed pN1 whereas 4 patients were called cN0 and subsequently confirmed pN0. In 6/9 men undergoing surgery the prostate cT-classification from PET/CT (mainly cT3b) was confirmed by final histopathology. One patient did not undergo surgery because of presence of nodal metastases above the aortic bifurcation and bone metastasis (cN1 cM1b).

### Intraoperative fluorescence guidance specifically delineates tumor tissue

After preoperative PET/CT imaging, patients underwent radical resection of the primary tumor including lymph node dissection or lymphadenectomy surgery. Nine patients (six laparoscopic, three open), mean age 64.9±7 years, BMI of 28.6±2.7 [kg/m^2^]), all ASA physical status classification system 3 with at least three primary diseases (arterial hypertension, coronary artery disease, chronic renal insufficiency, gout, diabetes, depression, obesity) and up to six long-term medications (ACE inhibitors, AT2 antagonists, allopurinol, insulin, antidepressants, statins) were treated. The mean duration of anaesthesia was 232.5±24.3 min, and the Post-anesthesia care unit stay was 303.1± 215 min (one patient stayed overnight). Throughout the entire anaesthetic management, none of the patients showed serious complications or unusual events. The surgical procedures were performed according to the state of the art with fluorescence guidance as an additional real-time information without changing the clinical routine process. Our retrospective analysis focused on describing our initial experiences concerning the intraoperative fluorescence signal and its dependence on the injected ligand dose. The fluorescence signal was classified according to the judgment of the surgeon on a scale of 0-2 (0: no fluorescence signal, 1: weak fluorescence signal, signal not strong enough to delineate cancer, 2: clear fluorescence signal, high suspicion for cancer with delineation from healthy tissue). While no fluorescence signal could be detected from the PET dose of the previous day and 200 µg (scale 0), a faint fluorescence signal was visualized in the primary tumor after application of 500 µg (scale 1). However, the signal was too weak to precisely identify and delineate tumor tissue in the prostate. Doses of 1000 µg and 1500 µg/patient of PSMA-914 resulted in a clear fluorescent signal (scale 2) at the primary tumor site with both, the conventional endoscopic camera and DaVinci firefly detection systems, respectively.

In addition, the fluorescent signal of PSMA-914 allowed for a rough assessment of the prostatic tumor location from outside the prostate as well as a precise visualization of the tumor extension from the prostate into the seminal vesicles (Figures 2 and 3, Videos S1 and S2). This was of particular benefit to the surgeon during preparation of the primary tumor. In cases of locally advanced prostate cancer lymph node metastases showed a clear fluorescence signal *in situ* that was consistent with suspicious palpation (Figures 2 and 3, Video S3). Overall, the fluorescence signal provided a high contrast to surrounding tissue and blood vessels for both primary tumors and lymph node metastases. Fluorescence in the urinary tract due to renal excretion of the hybrid molecule did not interfere with fluorescence guidance in the surgical field. *In situ* fluorescence findings were verified *ex situ* in the operation room in real time, confirming the intraoperative results (Figures S2 and S3).

### Postoperative imaging confirms PSMA-specific fluorescence

Histopathology and *ex situ* fluorescence imaging with the Odyssey CLx system agreed well, confirming the PSMA-specificity of the PSMA-914 fluorescence signal on the macroscopic level (Figures 2 and 3, Figure S4).

**Figure 4:**
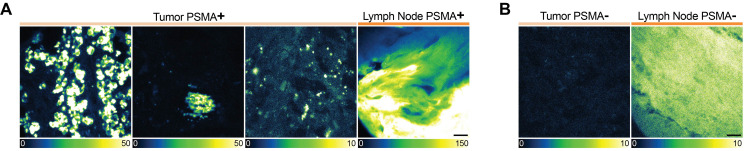
***Ex situ* confocal microscopy of PSMA-914.** Exemplary confocal images of PSMA-914 in (A) PSMA-positive and (B) PSMA-negative regions of tumor (left) and lymph node (right) tissue slices of one patient example (# 7). Raw data (counts) are shown; note the different look-up table scaling. Scale bar 10 μm.

To finally map the PSMA-914 fluorescence signal on the microscopic level, sets of representative *ex situ* confocal images of PSMA-positive and PSMA-negative tumor and lymph node regions were systematically acquired as per the histopathology, and the mean PSMA-914 fluorescence intensity per image was analyzed. In good agreement with the Odyssey CLx data, the PSMA-positive regions mostly showed a significantly higher mean PSMA-914 fluorescence intensity than the PSMA-negative regions per patient sample. However, the data of PSMA-positive regions did not correlate with the administered dose (Figure S5A), and the data of PSMA-negative regions and of the zero-dose patient did not show a clear baseline (Figure S5B). Moreover, the mean PSMA-914 fluorescence intensity varied remarkably for each patient sample and differed greatly between patient samples of the same dose. These observations reflect the great heterogeneity in the PSMA-914 distribution on the cellular level (Figure 4), which most likely emanates from a very heterogeneous cell population with a wide range of PSMA-expression levels 24.

## Discussion

The current study evaluates the novel PSMA-targeting peptidomimetic hybrid molecule, PSMA-914, for its use in PET imaging and fluorescence-guided surgery (FGS) in prostate cancer patients. This approach leverages the dual modality of PSMA-914, which combines positron emission tomography (PET) and near-infrared fluorescence (NIRF), to enhance tumor localization and resection. PSMA-914 offers several advantages over traditional imaging and surgical guidance techniques. Traditional blue dye and radioactive tracers used in sentinel lymph node biopsies often lack the precision and real-time feedback provided by NIRF imaging. In addition, the dual-modality nature of PSMA-914 allows for seamless integration of diagnostic imaging and surgical guidance, which is a significant advancement over single-modality tracers.

Our preliminary findings demonstrate that PSMA-914 is effective in preoperative PET/CT imaging for the detection of primary tumors and metastases, showing high tracer uptake in prostate cancer lesions. Further studies need to validate the diagnostic merits of PSMA-914 in comparison with other clinically established PSMA-targeting radiopharmaceuticals. The PET/CT imaging results were corroborated by post-operative histopathological evaluations, confirming the accuracy of PSMA-914 in staging lymph node involvement.

Intraoperatively, PSMA-914 provided a clear fluorescent signal that significantly aided the surgical resection process, if the injected doses exceeded 1000 µg/patient. At this dose both the conventional endoscopic camera and DaVinci firefly detection systems proved adequate for real-time visualization. This precise delineation is critical in achieving complete tumor resection while sparing healthy tissue, thus potentially improving surgical outcomes and reducing recurrence rates. Albeit these findings of a suitable dose for intraoperative fluorescence imaging are consistent with recently reported optimal doses of 25 µg/kg in a study with IS-002, a PSMA-targeting monomodal fluorescence imaging agent 25, further studies are needed to define the optimal dose. Concurrently, Chen *et al*. recently reported a first-in-human experience with [^68^Ga]Ga-P3, another dual-modality PSMA-targeting probe combining PET/CT and intraoperative fluorescence guidance in 16 patients undergoing RARP^26^. In contrast to PSMA-914, which was evaluated across a broader patient population including locally advanced and oligometastatic disease using both open and robotic surgical approaches, [^68^Ga]Ga-P3 was administered as a single combined dose 24 h prior to surgery exclusively in patients with localized prostate cancer. These complementary approaches underline the growing clinical interest in dual-modality PSMA-targeted probes and highlight the need for prospective comparative studies.

The specificity of PSMA-914-derived fluorescence was confirmed by histopathology and *ex vivo* fluorescence imaging, which correlated well with the PET/CT findings. However, the depth at which the fluorescence signal can be detected remains a concern. Current data do not provide sufficient insight into the effect of tissue thickness on the visibility of the fluorescence signal. For example, deep lymph node metastases covered by substantial adipose tissue may not be detectable with the current fluorescence intensity and resolution.

The safety profile of PSMA-914 was confirmed by preclinical toxicity studies, which showed no adverse effects at clinically relevant doses. In addition, the intraoperative use of PSMA-914 did not result in any significant complications or unusual events, indicating that it is well tolerated in the surgical setting. This is consistent with the safety data from similar PSMA-targeting agents used in diagnostic imaging and radioguided surgery.

While the results are promising, this retrospective analysis has several limitations. The sample size was small, and the study was conducted at a single center, using a pragmatic, clinically driven dose selection. Furthermore, a meaningful correlation analysis between preoperative SUVmax and intraoperative fluorescence signal was not feasible in this cohort, given the limited sample size, heterogeneous dosing, and use of different intraoperative imaging systems. This limits the generalizability of the findings. Future studies should include larger, multi-centric populations to validate the efficacy and safety of PSMA-914 in different patient populations. Additionally, long-term follow-up is needed to evaluate the impact of PSMA-914-guided surgery on patient outcomes, including recurrence rates and overall survival. The efficacy of fluorescence-guided surgery in terms of resolution and the ability to detect small residual tumor requires further investigation. The potential development and use of new fluorophores may improve the brightness and resolution, allowing for the detection of smaller and less PSMA-expressing cancerous tissue.

In summary, PSMA-914 represents a significant advancement in the field of fluorescence-guided surgery for prostate cancer. Its ability to provide real-time, high-contrast visualization of tumor tissue during surgery has the potential to improve surgical precision and, consequently, patient outcomes. The promising results of this study underscore the need for further clinical trials not only to validate the efficacy and safety of PSMA-914 in larger patient cohorts. Investigations to optimize the fluorophore properties, improve detection technologies, and integrate multimodal imaging techniques will be critical. In addition, regulatory approval for the use of compatible endoscopes and imaging devices will be required to translate this theranostic approach into routine clinical practice.

## Conclusion

Our first experiences evaluate PSMA-914, a novel PSMA-targeting hybrid molecule, and demonstrate its efficacy in preoperative PET imaging and intraoperative fluorescence-guided surgery (FGS) for prostate cancer. PSMA-914-PET/CT achieved high diagnostic accuracy in the pretherapeutic staging of prostate cancer, correctly classifying nodal status in all patients and confirming the local tumor stage in the majority. By combining positron emission tomography and near-infrared fluorescence imaging, PSMA-914 may provide precise tumor localization and real-time surgical guidance. Clinical integration of PSMA-914 could enhance surgical precision, reduce positive margin rates, and ultimately improve patient outcomes. Larger prospective studies with direct comparisons to established PSMA-targeted agents are warranted to validate these promising initial results and demonstrate a benefit for patient treatment.

## Supplementary Material

Supplementary methods and figures.

Supplementary videos.

## Figures and Tables

**Table 1:** Preoperative information and data from [^68^Ga]Ga-PSMA-914 PET/CT.

Patient N°	Age at PET/CT [years]	PSA^1^ at PET/CT [ng/ml]	Gleason-score (biopsy)	Activity at PET/CT [MBq]	Time between PET/CT and surgery [days]	Clinical staging based on PET/CT		
						cT-stage	cN-stage	cM-stage
1	67	23.0	4+5 (ISUP5)	197	1	cT3b (both sides)	cN1	cM1a, b
2	73	13.0	4+5 (ISUP5)	118	1	cT3b (right)	cN0	cM0
3	60	3.68	4+4 (ISUP4)	211	1	cT3	cN1	cM0
4	70	9.57	4+5 (ISUP5)	217	1	cT2c	cN0	cM0
5	72	7.0	4+5 (ISUP5)	205	1	cT3b (left)	cN1	cM0
6	56	6.41	4+5 (ISUP5)	210	1	cT3	cN0	cM0
7	76	99.0	5+4 (ISUP5)	195	6	cT3b	cN1	cM0
8	54	140	4+5 (ISUP5)	206	6	cT3b (both sides)	cN0	cM1b
9	58	21.6	4+5 (ISUP5)	187	-	cT3b(right)	cN1	cM1a,b
10	65	2.95	3+4 (ISUP2)	211	46	cT3b (both sides)	cN1	cM0

^1^ PSA = Prostate specific antigen

**Table 2:** Postoperative findings and intraoperative fluorescence detection compared with preoperative PET/CT results

Patient N°	Surgical technique of radical prostatectomy	cold PSMA-914 dose	Pathological staging form final histology							Detection of intraoperative fluorescence**		SUV_max_ (g/mL) in PET/CT 1h post injection	
			Final Gleason-score	pT-stage	pN-stage^2^	L-stage^3^	V-stage^4^	Pn-stage	R-stage^5^	Prostate	Lymph nodes	Prostate	Lymph node
1	RARP^1^	-*	4+5 (ISUP5)	3b (both sides)	1 (4/17)	1	0	1	1	negative (0)	negative (0)	8.7	26.0
2	RARP	200 µg	4+3 (ISUP3)	3b (right)	0 (0/11)	0	0	1	1	negative (0)	negative (0)	25.1	NA
3	RARP	500 µg	4+4 (ISUP4)	3a	1 (1/16)	1	1	0	0	weak (1)	negative (0)	5.9	9.2
4	RARP	-^†^	4+5 (ISUP5)	3b (left)	0 (0/21)	0	0	1	0	-	-	5.9	NA
5	RARP	1000 µg	4+5 (ISUP5)	3b (left)	1 (3/32)	1	1	1	0	positive (2)	weak (1)	10.0	5.9
6	RARP	1500 µg	4+4 (ISUP4)	3a	0 (0/8)	0	0	1	0	positive (2)	negative	6.3	NA
7	retropubic open	1000 µg	5+4 (ISUP5)	4	1 (7/39)	1	0	1	1	positive (2)	positive (2)	65.2	15.1
8	retropubic open	1000 µg	4+5 (ISUP5)	3a	0 (0/39)	0	0	1	0	positive (2)	negative	15.5	NA
9	No surgery due to extensive metastatic disease	-	-	-	-	-	-	-	-	-	-		
10	retropubic open	1000 µg	4+3 (ISUP3)	3b (both sides)	1 (2/42)	0	0	1	1	positive (2)	positive (2)	6.7	4.2

^1^RARP = robot-assisted radical prostatectomy, ^2^Pn = perineural invasion, ^3^L = lymphangiosis, ^4^V = hemangiosis carcinomatosa, ^5^R = resection margin, *surgery without additional cold PSMA-914 administration; intraoperative fluorescence imaging was performed using the residual tracer from the preoperative [^68^Ga]Ga-PSMA-914 PET/CT dose the day before surgery (30 µg). ^†^ surgery without fluorescence imaging due to a technical failure of the intraoperative fluorescence imaging system. **scale fluorescence signal: 0 = no fluorescence signal, 1= weak fluorescence signal, signal not strong enough to delineate cancer, 2 = clear fluorescence signal, high suspicion for cancer with delineation from healthy tissue, NA = not applicable
