# Supplementary material for: PSMA-targeting hybrid molecule PSMA-914 for preoperative imaging and intraoperative fluorescence-guidance: A first-in-human experience
Source: Theranostics. 2026 Jul 5;16(14):7926–36. doi: 10.7150/thno.129889 (PMC13440268; doi:10.7150/thno.129889)
Supplement: Supplementary file 1 [file thnov16p7926s1.pdf]

Supplemental Information

PSMA-targeting hybrid molecule PSMA-914 for preoperative imaging and intraoperative fluorescence-guidance: A first-in-human experience

Ann-Christin Eder[1,2,*], Mohamed A. Omrane[1,2], Jessica Matthias[3, #], Sven Stadlbauer[4,5,6], Mareike Roscher[4,7], Kerstin Michalski[1f], Martin A. Schick[8], Christoph-Ferdinand Wielenberg[1], Peter Bronsert[9], Christian Gratzke[10], Martin Freitag[1], Klaus Kopka[4,5,6,11], Matthias Eder[1,2], Philipp T. Meyer[1,12], Cordula A. Jilg[10‡], Juri Ruf[1,12,†, ‡]

[1] Department of Nuclear Medicine, Medical Center - University of Freiburg, Faculty of Medicine, University of Freiburg, Freiburg, Germany

[f] present address: Department of Nuclear Medicine, University Hospital Würzburg, Würzburg, Germany

[2] Division of Radiopharmaceutical Development, German Cancer Consortium (DKTK), partner site Freiburg, Freiburg, Germany and German Cancer Research Center, Heidelberg, Germany

[3] Department of Optical Nanoscopy, Max Planck Institute for Medical Research, Heidelberg, Germany

[#] present address: Abberior Instruments America, Bethesda, USA

[4]Division of Radiopharmaceutical Chemistry, German Cancer Research Center (DKFZ), Heidelberg, Germany

[5]Helmholtz-Zentrum Dresden-Rossendorf (HZDR), Institute of Radiopharmaceutical Cancer Research, Dresden, Germany

[6]TUD Dresden University of Technology, School of Science, Faculty of Chemistry and Food Chemistry, Dresden, Germany

[7]Service Unit for Radiopharmaceuticals and Preclinical Studies, German Cancer Research Center (DKFZ), Heidelberg, Germany

[8]Department of Anesthesiology and Critical Care, Medical Center–University of Freiburg, Faculty of Medicine, University of Freiburg, Freiburg, Germany

[9]Department of Pathology, Medical Center - University of Freiburg, Faculty of Medicine, University of Freiburg, Freiburg, Germany

[10]Department of Urology, Medical Center – University of Freiburg, Faculty of Medicine, University of Freiburg, Freiburg, Germany

[11]German Cancer Consortium (DKTK), partner site Dresden, Dresden, Germany

[12]German Cancer Consortium (DKTK), partner site Freiburg, Freiburg, Germany

[†] present address: Klinikum Karlsruhe, Department of Nuclear Medicine, Karlsruhe, Germany

* Corresponding author:
Dr. Ann-Christin Eder
Department of Nuclear Medicine, University Medical Center Freiburg and Division of Radiopharmaceutical Development, German Cancer Consortium, partner site Freiburg, and German Cancer Research Center, Heidelberg, Germany
Hugstetter Str. 55
79106 Freiburg
Tel: +49 761 270 74221
Fax: +49 761 270 39980

Supplemental Methods 3

Supplemental Figures 9

Supplemental Figure 1. Schematic overview of automated radiopharmaceutical preparation of [^{68}Ga]Ga-PSMA-914. 10
Supplemental Figure 2. Example of *ex situ* real-time fluorescence imaging of tumorous prostate after robot-assisted radical prostatectomy. 10
Supplemental Figure 3. Example of *ex situ* real-time fluorescence imaging after retropubic open prostatectomy with lymph node dissection. 11
Supplemental Figure 4. *Ex situ* fluorescence imaging and PSMA-specific staining after retropubic open prostatectomy with lymph node dissection. 12
Supplemental Figure 5. Quantitative confocal image analysis of *ex vivo* resected tissue data. 12

References 12

Supplemental Methods

Radiopharmaceutical Preparation of [^{68}Ga]Ga-PSMA-914

The radiopharmaceutical [^{68}Ga]Ga-PSMA-914 was prepared in a GMP grade C environment using fully automated GMP-compliant synthesis module (Eckert & Ziegler) with single use sterile cassettes. [^{68}Ga]$GaCl_3$ eluted from ^{68}Ge/^{68}Ga generator (GalliaPharm®, Eckert & Ziegler) was pre-concentrated on strong cation exchange cartridge (SCX) and eluted with 0.3 – 0.5 mL SCX-solution (5 M NaCl / 5.5 M HCl) to the reaction vial containing 31 µg PSMA-914, 0.3 ml sodium acetate / HCl buffer (pH 4.5), 1.0 ml water for injection and 50 mg ascorbic acid. The radiolabeling was performed at 95 °C for 300 seconds. The reaction mixture was cooled down, diluted with saline and purified with a preconditioned Sep-Pak® light C8 cartridge. The product was eluted with 70 % ethanol, diluted with 7 ml saline and 0.5 ml ascorbic acid for injection (100 mg / ml). The final formulated product was sterile filtrated by passing the product through a 0.22-µm sterile filter. The integrity test of the filter was performed immediately after the final production step. Subsequently aliquots were withdrawn for quality control and sterility testing. The quality control process included pH test by pH strip, bacterial endotoxins by chromogenic LAL-test method, radionuclide identity and purity test by determining half-life and energy spectrum. Chemical and radiochemical purity (≥ 95 %) were identified by radio-high-performance liquid chromatography and radio instant thin layer chromatography (radio-iTLC). The residual solvent impurities were determined by gas chromatography and the sterility tests were performed in independent laboratories (Biochem, Karlsruhe, Germany) according to Ph. Eur. and USP.

Safety, Tolerability and Pharmakokinetics

Methods

The toxicology and dosimetry study was monitored by ABX-CRO advanced pharmaceutical services Forschungsgesellschaft mbH (Dresden, Germany) and the studies undertaken at LPT Laboratory of Pharmacology and Toxicology GmbH & Co. KG (Hamburg, Germany) and the Department of Preclinical Imaging and Radiopharmacy of University Hospital Tübingen (Tübingen, Germany), respectively. The animal experiments were approved by the responsible local authorities in compliance with the current laws of the Federal Republic of Germany.

For the subchronic toxicity study, 52 days old male CD-1 mice (Charles River Laboratories, Sulzfeld, Germany; n=12) were injected once daily for 14 days with 0, 0.2, 1 and 2 mg/kg body weight PSMA-914 as i.v. bolus in a tail vein (administration volume 10 mL/kg body weight/day). In the low-dose group, six additionally treated mice were monitored during a subsequent two-week recovery period. PSMA-914 was dissolved in PBS as stock solution, sterile filtered and stored in aliquots at -20° C until the day of use. All mice were housed singly under standard conditions in approved facilities with 12-h light/dark cycles and given food and water *ad libitum*. The mice in the different treatment groups were allocated by means of a computer-generated randomization program (Provantis® Integrated preclinical software, version 10.2, Instem LSS Ltd., Stone, Staffordshire ST15 0SD, United Kingdom). The experiment was conducted according to the rules of GLP.

The following parameters were monitored:

- clinical signs: skin/fur, eyes, mucous membranes, respiratory and circulatory systems, somatomotor activity and behaviour patterns, morbidity; special attention to local tolerance at injection site
- body weight
- food and drinking water consumption
- haematology: hemoglobin content (HGB), erythrocytes (RBC), leucocytes (WBC), reticulocytes (Reti), platelets (PLT), hematocrit value (HCT), mean corpuscular volume (MCV), mean corpuscular haemoglobin (MCH), mean corpuscular haemoglobin concentration (MCHC). Haematological analysis was undertaken with an ADVIA™ 120 (Siemens Diagnostics GmbH, Fernwald, Germany).
- clinical biochemistry: bilirubin (total), cholesterol, creatinine, glucose, protein (total), urea (in blood), calcium, chloride, potassium, sodium, alanine amino-transferase (ALAT), alkaline phosphatase (aP), aspartate aminotransferase (ASAT), lactate dehydrogenase (LDH). Analysis was performed via KONELAB 30i (Thermo Fisher Scientific, Dreieich, Germany).

- urine analysis: volume, pH, specific gravity; protein, glucose, bilirubin, urobilinogen, ketones, haemoglobin (Hb, approx. values), nitrite; epithelial cells, leucocytes, erythrocytes, organisms, further constituents (i.e. sperm, casts), crystalluria
- macroscopy, organ weights, and histopathology.

The µPET/MR radiation dosimetry study was performed using seven week old C57BL/6J mice (Charles River Laboratories, Sulzfeld, Germany; n=6 with each 3 males and 3 females). All mice were housed in groups under standard conditions in approved facilities with 12-h light/dark cycles and given food and water *ad libitum*. The mice were injected i.v. with 12.29 (±0.47) MBq [^{68}Ga]Ga-PSMA-914 and ~0.6 µg (~205 pmol; specific acitivity in a range of 21.15-21.92 MBq/µg) PSMA-914 (RCP ≥98%) on three different days (pairs of each one male and one female).

Starting from the injection, a dynamic PET image was acquired in list mode over 3 hours, followed by a 15 min attenuation scan and anatomical MR imaging using a T2 turbo-RARE sequence. For PET imaging, a Siemens Focus 120 micro PET (Siemens Medical Solutions Inc.); for MRI a Bruker BioSpec 70/30 USR MR scanner (Bruker Biospin, Ettlingen, Germany) was used.

After completion of the imaging (~4h p.i.), animals were sacrificed and organs taken for an *ex vivo* biodistribution (n=4). Selected tissues and organs were collected, weighed, and measured in a gamma counter (Wizard 2480, PerkinElmer, Rodgau, Germany). The results were calculated as percentage of the injected dose per gram of tissue mass (% ID/g) after decay correction to injection time. For the kidneys, values are given as %ID/organ.

PET data were reconstructed using an unweighted 2D OSEM algorithm with a final pixel size of (X, Y, Z) = 0.4/0.4/0.8 mm, a zoom factor of 1, and framed as 6 x 10 s, 9 x 60 s, 4 x 300 s, 5 x1800 s. Images were normalized and corrected for attenuation, dead-time, and physical decay. Co-registration and data analysis for the respective volumes of interest (VOI) and subsequent (non-)decay corrected time-activity curves (TACs) were performed in PMOD v4.0 (PMOD Technologies, Fällanden, Switzerland).

Based on the image analysis and the decay-corrected TACs, information for pharmacokinetics (i.e. biodistribution characteristics) of [^{68}Ga]Ga-PSMA-914 were extracted. The apparent biological half-life of blood clearance was determined by fitting a bi-exponential function to the generated decay-corrected blood pool TACs. The longer time component of the bi-exponential function represents the apparent biological half-life while the shorter time component (kept under 5 minutes in all cases) allows a better description of the blood distribution and clearance. Similarly, a mono-exponential function was fitted to the generated decay-corrected bladder

TAC for each mouse to determine the half-life of the bladder filling, which is representative of the mouse renal excretion.

For allometric calculations which were performed at ABX CRO, the generated non-decay-corrected organ TACs were scaled from mice to humans according to Constantinescu et al. (2013) assuming the weight of an average man (body mass = 73 kg, height = 1.76 m) and an average woman (body mass = 60 kg, height = 1.63 m) according to Bolch et al. (2016) [1, 2]. Body weight and organ masses of the mice were obtained from the *ex vivo* biodistribution study. The scaled human TACs were incorporated in the CE-marked dose calculation software QDOSE v 1.1.10 (ABX-CRO advanced pharmaceutical services Forschungsgesellschaft mbH, Dresden, Germany). In QDOSE, the scaled human TACs were fitted to a sum of exponential functions and further integrated and normalized to generate time-integrated activity coefficient (TIAC) values (formerly called residence times).

<u>Statistics</u>

For the toxicity study, the data obtained from the treated groups were compared with the control group. Homogeneity of variances and normality of distribution for body weight, food consumption, haematology, clinical chemistry, urine analysis (spec. gravity, pH, volume), relative, and absolute organ weights were tested using BARTLETT's test and the SHAPIRO-WILK's test. In case of heterogeneity and/or non-normality of distribution, stepwise transformation of the values into logarithmic or rank tvalues was performed prior to ANOVA. If the ANOVA yielded a significant effect ($p \leq 0.05$), intergroup comparisons with the control group were made by DUNNETT's test. Histology data were analysed using FISHER`s exact test.

<u>Results</u>

For the subchronic toxicity study, male mice were injected daily with 0, 0.2, 1 or 2 mg/kg body weight PSMA-914 for a period of two weeks. Dose levels were chosen based on the microdose approach. A 1000 time higher dose than the human one (i.e. 100 mg/50 kg body weight) was selected (2.0 mg/kg body weight), with subsequent groups of 50% (1.00 mg/kg, intermediate dose) and 10% (0.200 mg/kg, low dose) of the high dose level. None of the animals died or had to be sacrificed due to moribundancy. Importantly, no local intolerance reactions at the injection sites or changes in behaviour and external appearance were detected. Furthermore, also for the other monitored parameters (body weight and body weight gain, food and drinking water consumption, haematology, urine analysis, organ weights) no adverse effects could be observed. The macroscopic inspection of the organs for necropsy and histopathology of organs and tissues showed no clinical signs after treatment. Also, for the animals which were monitored in a two week recovery period after treatment with 0.2 mg/kg body weight PSMA-

914, no adverse effects could be monitored. Variations are considered to be within the limits of normal biological variability or due to spontaneous changes (e.g. mouse 33, intermediate dose, test day 15, presented with a left kidney reduced in size). In conclusion, the no-effect-observed level (NOEL) is above 2.0 mg/kg body weight PSMA-914 by daily i.v. administration.

For the µPET radiation dosimetry study, PET/MR data were acquired after injection of [^{68}Ga]Ga-PSMA-914 in a healthy mouse model followed by an *ex vivo* organ biodistribution study. The biodistribution profile and uptake of the radiotracer were determined and the data used for allometric modelling of radiation dosimetry in healthy human tissue.

The dynamic PET/MR imaging of [^{68}Ga]Ga-PSMA-914 over 3 hrs and the time activity curves generated from the individual frames revealed the renal system as the main excretion route since the radiotracer accumulated mainly in the kidneys and subsequently also in the bladder. No other organ showed a specific uptake of [^{68}Ga]Ga-PSMA-914 neither in male or female mice. The overall biological half-life blood clearance was 1.2 (±0.29) hours. For the kidneys, the half-life could not be calculated since the radiotracer did not reach a plateau or start decreasing by the end of the image acquisition.

The data of the *ex vivo* biodistribution study 4h p.i. of [^{68}Ga]Ga-PSMA-914 are comparable to the results obtained for the µPET imaging. While the clearance from blood was almost complete and only low uptake in the heart, spleen and liver could be observed, respectively, the uptake of the radioligand in the kidney as main excretion organ was still relatively high with around 20% ID/organ. Between the sexes no significant difference in the biodistribution of PSMA-914 was detectable.

Based on the acquired data from the healthy mice used for the radiation dosimetry, dose calculations for humans were performed using IDAC-Dose 2.1 for an average man and woman to estimate normalized absorbed organ doses and normalized effective dose. The normalized effective dose scaled for an average human (i.e. mean between average man and average woman) after administration of [^{68}Ga]Ga-PSMA-914 was 1.50×10^{-2} ($\pm 5.61 \times 10^{-4}$) mSv/MBq. Assuming a standard injected activity for diagnosis of 150 MBq per patient, an effective dose of 2.25 (±0.08) mSv can be expected for an average patient (mean between male and female). In addition, for the standard diagnostic activity, the kidneys are expected to receive 8.72 (±1.58) mGy for the average man and 10.29 (±1.86) mGy for the average woman.

Anaesthesia and Safety

Patients were scheduled for elective surgery and underwent SOP. For laparoscopic balanced anaesthesia and for laparatomic prostatectomies TIVA (Total Intravenous Anesthesia) were performed, using the following substances for anesthesia: sufentanil, remifentanil, piritramide, propofol, desflurane or sevoflurane, cisatracurium or rocuronium, norepinephrine, Akrinor (cafedrine/theodrenaline), cefazolin, metamizole, and dexamethasone. For all patients standard monitoring ECG, blood pressure measurement SPO_2, BIS and capnography were used. Long-term medications were discontinued or continued according to our guidelines. Postoperatively, all patients were send to the PACU (Post-Anaesthesia Care Unit) and were transferred upon achieving hemodynamic stability, sufficient respiration, and pain <3 on the VAS (Visual Analog Scale). Patients with obstructive sleep apnoea without devices stayed overnight in the PACU. Adverse events were monitored from the time of PSMA-914 administration until hospital discharge, encompassing the preoperative phase after tracer injection, intraoperative monitoring, and the entire postoperative in-hospital period. The mean duration of in-hospital observation was 7–8 days, resulting in an overall safety monitoring window of approximately 8–9 days following tracer administration.

Confocal Microscopy of Tissue Slices

Confocal data were acquired on a custom-built STED (Stimulated Emission Depletion) system similar to the one published by Gorlitz et al.[3]. A nanosecond pulsed 775 nm fiber laser (MPBC, usually employed as depletion laser for STED imaging) was used at 57 mW for excitation of PSMA-914, and a pulsed 470 nm diode laser (LDH-D-C470, PicoQuant) was used at 66 µW for excitation of tissue autofluorescence, with detection windows of 800-870 nm and 500-550 nm, respectively. Laser powers were measured in front of the microscope body.

The histopathological stainings were used as reference to macroscopically identify several PSMA-positive and PSMA-negative regions of interests on the individual tissue slices. To minimize user bias and account for potential microscopic tissue heterogeneity, a grid pattern of 80 µm x 80 µm sized fields of view was centred onto these regions of interest and confocal images were systematically acquired in line multiplexing mode with a pixel size of 100 nm and a dwell time of 100 µs without line accumulation.

The mean fluorescence intensity was extracted with a dedicated ImageJ script [4]. This routine generates a mask for each autofluorescence image based on a fixed signal intensity threshold distinguishing between tissue absence (set to “not-a-number”) and tissue presence (set to “1)”. Multiplying this mask with its corresponding raw PSMA-914 image creates an output image with which the mean PSMA-914 fluorescence intensity can be evaluated via ImageJ’s *Measure*

function exclusively in the relevant parts of the raw PSMA-914 image without summing up background from tissue-free sample regions.

Supplemental Figures

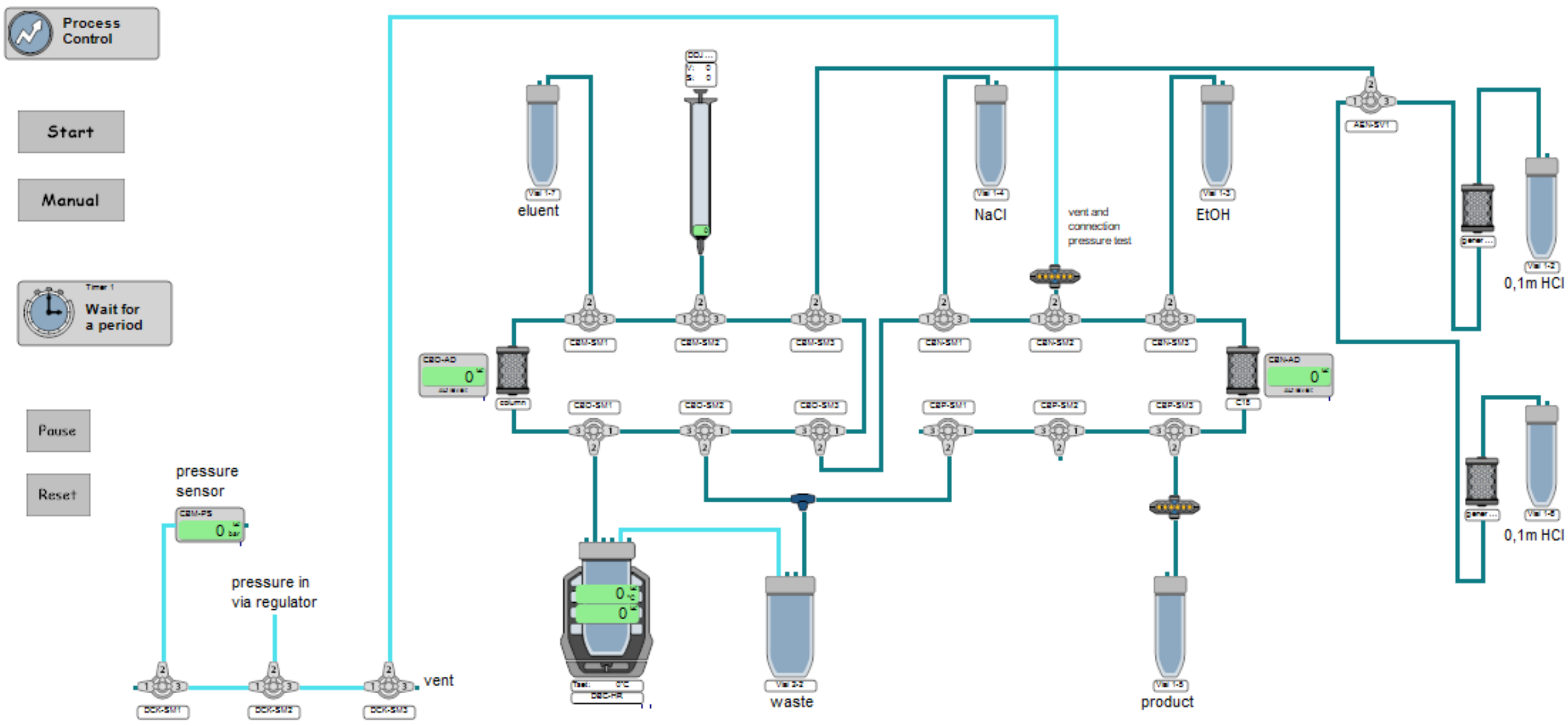

Supplemental Figure 1. Schematic overview of automated radiopharmaceutical preparation of [^{68}Ga]Ga-PSMA-914.

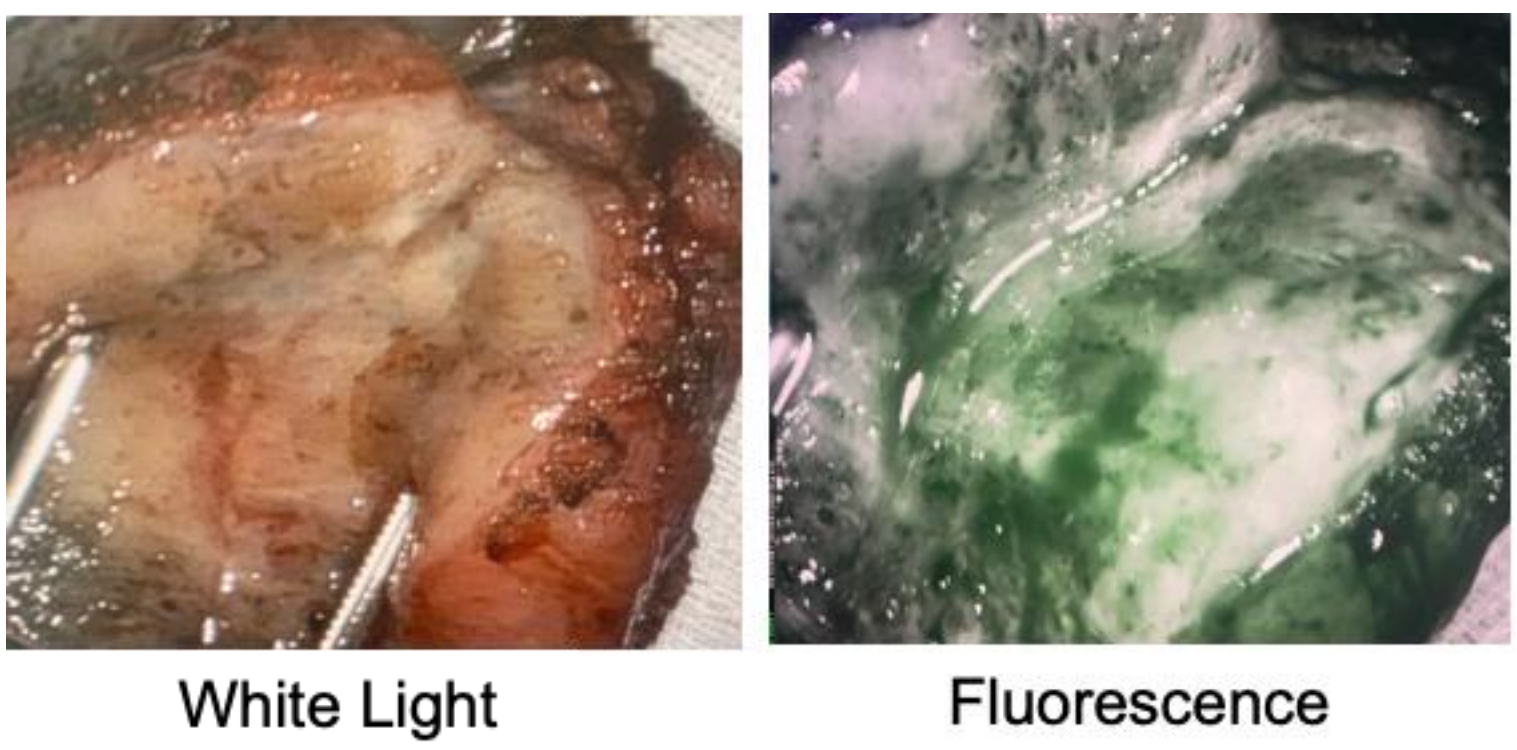

Supplemental Figure 2. Example of *ex situ* real-time fluorescence imaging of tumorous prostate after robot-assisted radical prostatectomy. Real-time fluorescence imaging of the bisected cancerous prostate (1000 µg PSMA-914) under white light (left) and fluorescence (right).

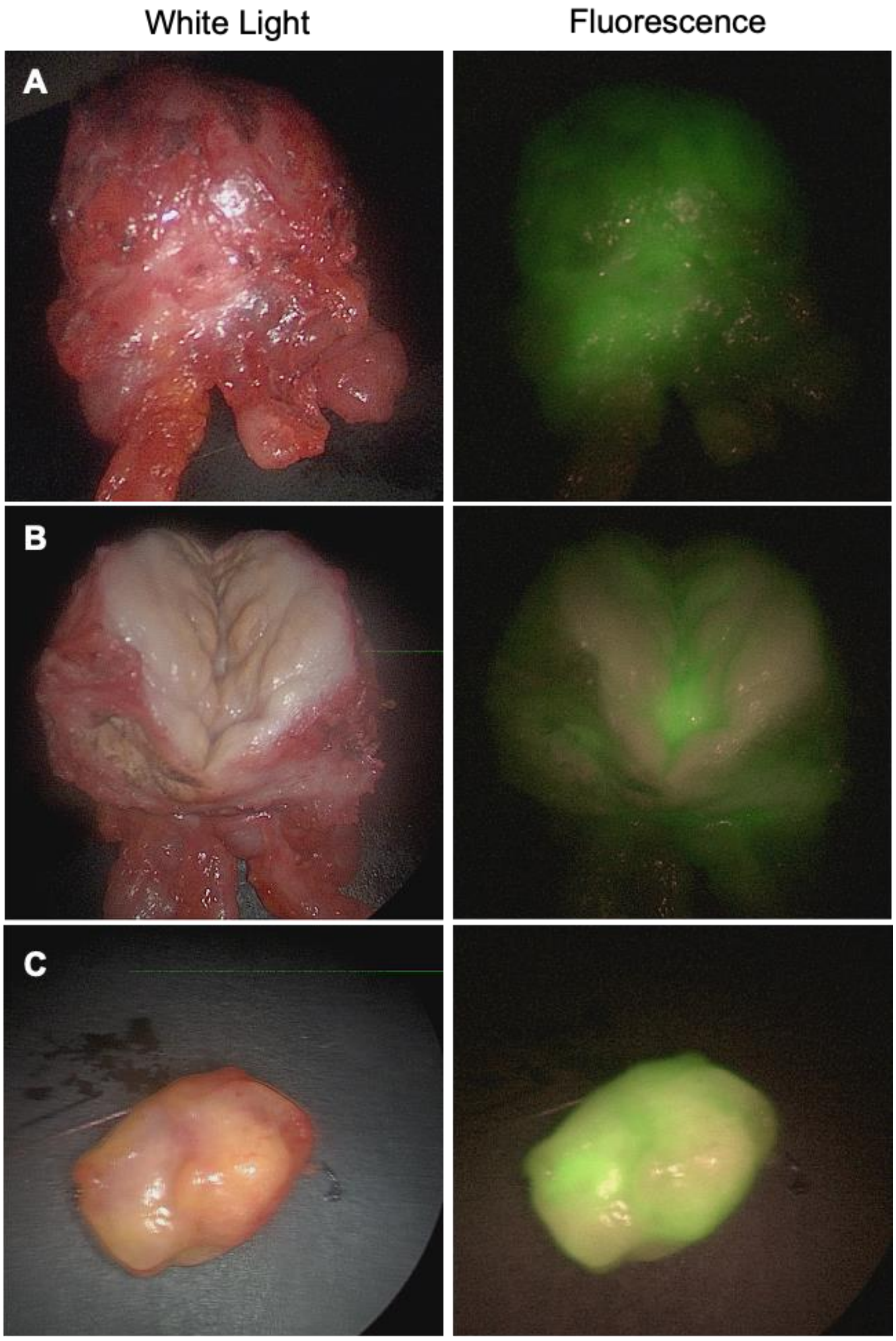

Supplemental Figure 3. Example of *ex situ* real-time fluorescence imaging after retropubic open prostatectomy with lymph node dissection. Real-time fluorescence imaging of the (A) cancerous prostate, (B) bisected cancerous prostate, and (C) dissected lymph node (1000 µg PSMA-914) under white light (left) and fluorescence (right).

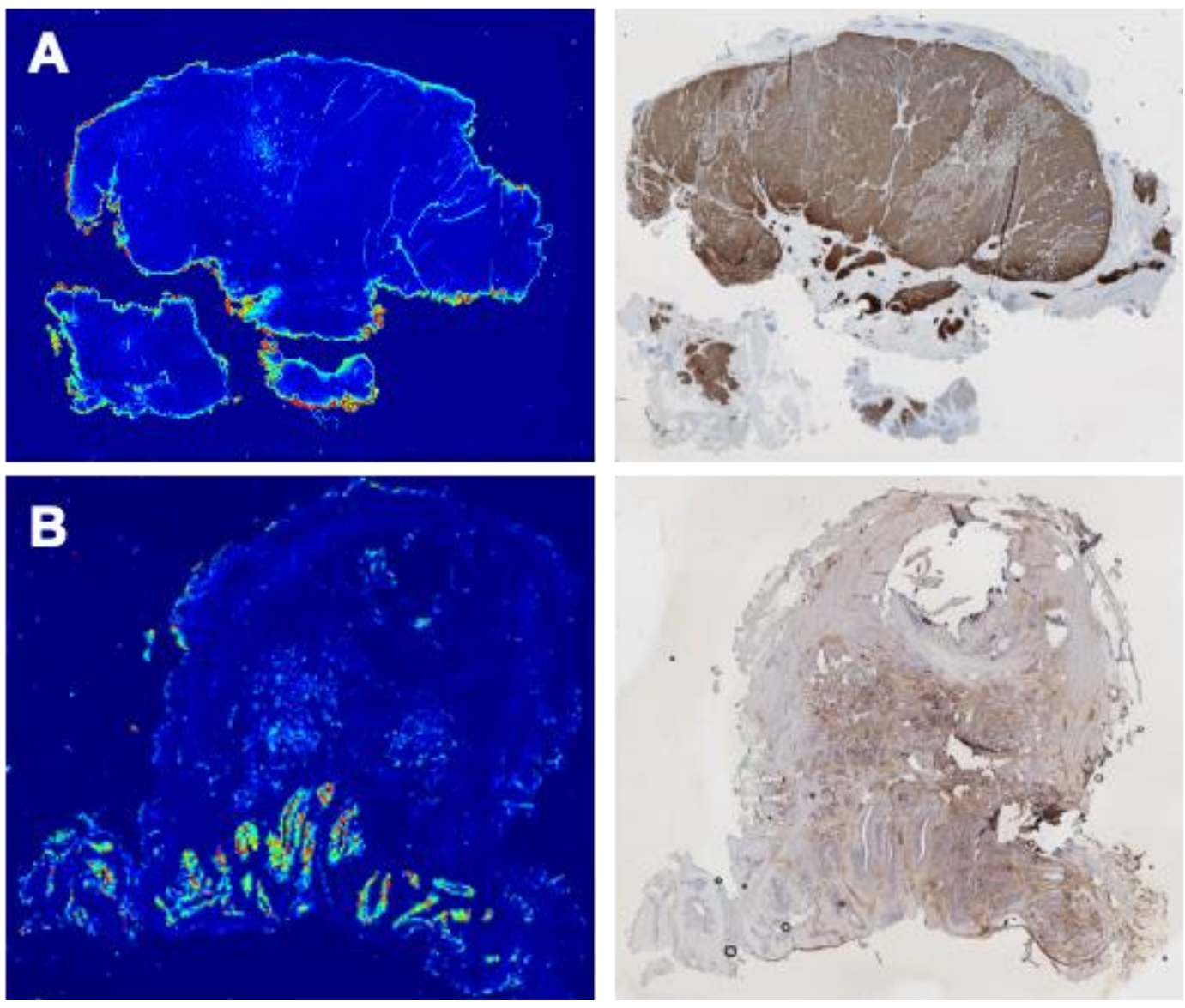

Supplemental Figure 4. ***Ex situ*** **fluorescence imaging and PSMA-specific staining robot-assisted radical prostatectomy with lymph node dissection.** Odyssey CLx fluorescence imaging (left) and PSMA-specific immunohistochemical staining (right) of a (A) dissected lymph node, and (B) cancerous prostate (preoperative [^{68}Ga]Ga-PSMA-914 PET/CT dose the day before surgery (30 µg)) as a fluorescence negative reference.

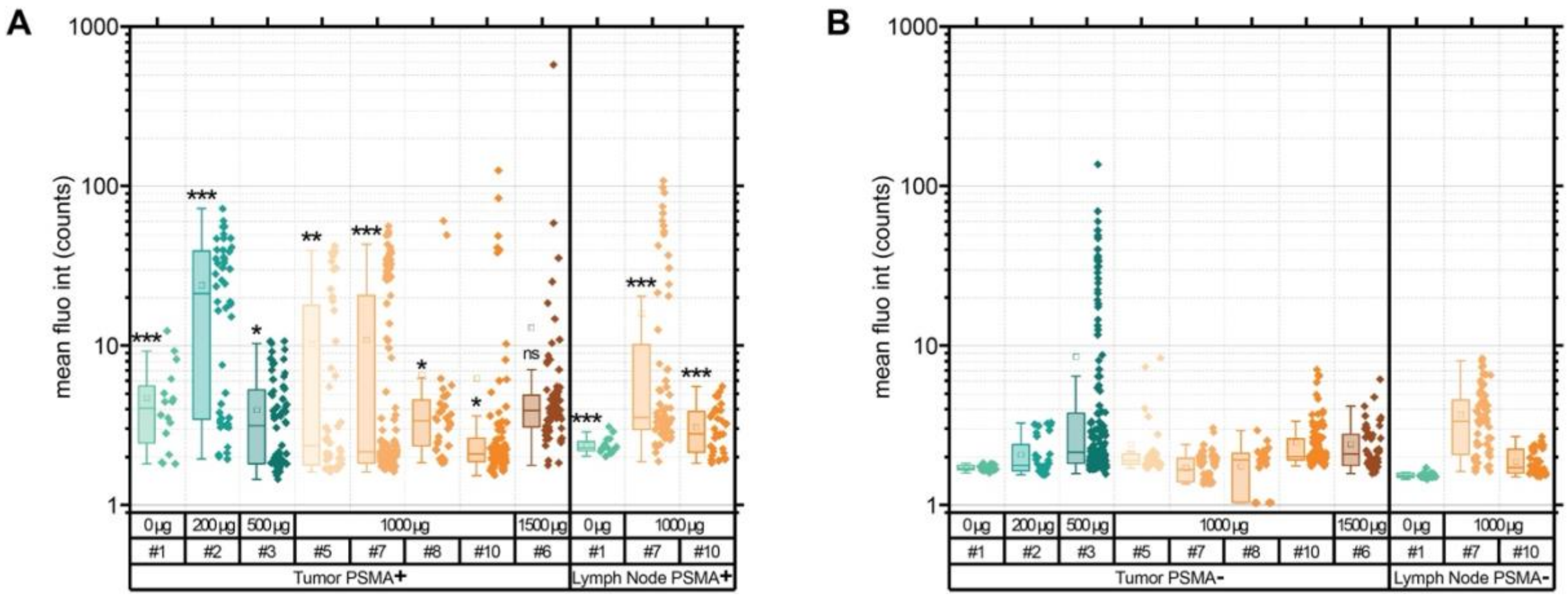

Supplemental Figure 5. Quantitative confocal image analysis of ***ex vivo*** **resected tissue data.** Mean fluorescence intensity in (A) PSMA-positive tumor regions and lymph nodes, and in (B) PSMA-negative tumor regions and lymph nodes across different PSMA-914 doses (0-1000 µg, #N - patients as indicated). Box plot shows the interquartile range (box), the outer-most data points falling within 1.5× interquartile range (whiskers), the median (center line), and the mean (square) of individual confocal tissue images per patient. Asterisks indicate statistical significance (*p<0.05, **p<0.005, ***p<0.005, ns – no significant differences).

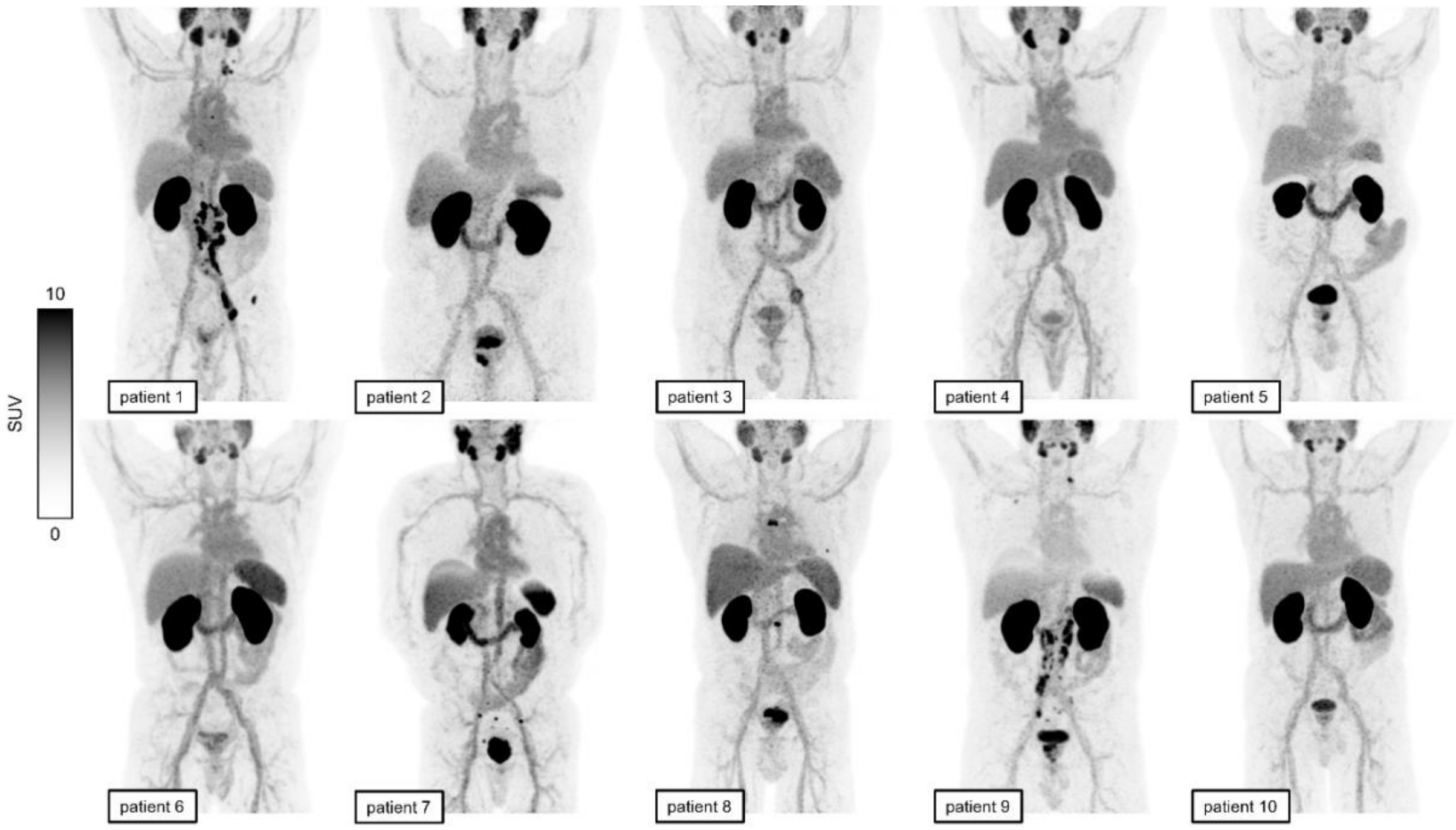

Supplemental Figure 6. Maximum intensity projections (MIPs) of all patients 1 hour after injection of [^{68}Ga]Ga-PSMA-914.

References

1. Constantinescu CC, Sevrioukov E, Garcia A, Pan ML, Mukherjee J. Evaluation of [18F]Mefway biodistribution and dosimetry based on whole-body PET imaging of mice. Mol Imaging Biol. 2013; 15: 222-9.
2. Bolch WE, Jokisch D, Zankl M, Eckerman KF, Fell T, Manger R, et al. ICRP Publication 133: The ICRP computational framework for internal dose assessment for reference adults: specific absorbed fractions. Ann ICRP. 2016; 45: 5-73.
3. Gorlitz F, Hoyer P, Falk H, Kastrup L, Engelhardt J, Hell SW. A STED Microscope Designed for Routine Biomedical Applications (Invited Paper). Progress In Electromagnetics Research. 2014; 147: 57-68.
4. Schneider CA, Rasband WS, Eliceiri KW. NIH Image to ImageJ: 25 years of image analysis. Nat Methods. 2012; 9: 671-5.
